# Metabolomes and Lipidomes of the Infective Stages of the *Gastrointestinal nematodes*, *Nippostrongylus brasiliensis* and *Trichuris muris*

**DOI:** 10.3390/metabo10110446

**Published:** 2020-11-06

**Authors:** Karma Yeshi, Darren J. Creek, Dovile Anderson, Edita Ritmejerytė, Luke Becker, Alex Loukas, Phurpa Wangchuk

**Affiliations:** 1 Centre for Molecular Therapeutics, Australian Institute of Tropical Health and Medicine, James Cook University, Building E4, McGregor Rd, Smithfield, Cairns, QLD 4878, Australia; edita.ritmejeryte@jcu.edu.au (E.R.); luke.becker@jcu.edu.au (L.B.); alex.loukas@jcu.edu.au (A.L.); 2 Drug Delivery, Disposition and Dynamics, Monash Institute of Pharmaceutical Sciences, Monash University, 381 Royal Parade, Parkville, VIC 3052, Australia; darren.creek@monash.edu (D.J.C.); dovile.anderson@monash.edu (D.A.)

**Keywords:** metabolites, infective stage, *Nippostrongylus brasiliensis*, *Trichuris muris*, parasites, LC-MS, metabolomic, lipidomic

## Abstract

Soil-transmitted helminths, including hookworms and whipworms, infect billions of people worldwide. Their capacity to penetrate and migrate through their hosts’ tissues is influenced by the suite of molecules produced by the infective developmental stages. To facilitate a better understanding of the immunobiology and pathogenicity of human hookworms and whipworms, we investigated the metabolomes of the infective stage of *Nippostrongylus brasiliensis* third-stage larvae (L3) which penetrate the skin and *Trichuris muris* eggs which are orally ingested, using untargeted liquid chromatography-mass spectrometry (LC-MS). We identified 55 polar metabolites through Metabolomics Standard Initiative level-1 (MSI-I) identification from *N. brasiliensis* and *T. muris* infective stages, out of which seven were unique to excretory/secretory products (ESPs) of *N. brasiliensis* L3. Amino acids were a principal constituent (33 amino acids). Additionally, we identified 350 putative lipids, out of which 28 (all known lipids) were unique to N. brasiliensis L3 somatic extract and four to *T. muris* embryonated egg somatic extract. Glycerophospholipids and glycerolipids were the major lipid groups. The catalogue of metabolites identified in this study shed light on the biology, and possible therapeutic and diagnostic targets for the treatment of these critical infectious pathogens. Moreover, with the growing body of literature on the therapeutic utility of helminth ESPs for treating inflammatory diseases, a role for metabolites is likely but has received little attention thus far.

## 1. Introduction

Infection with parasitic helminths is one of the most common and detrimental of the neglected tropical diseases [1]. Indeed, eight out of the 17 recognised neglected tropical diseases are caused by parasitic helminths [2]. More than 1.5 billion people (approximately 24% of the world’s population) are infected with soil-transmitted helminth infections (STHIs) [3] and contribute to a substantial burden of human disease and disability worldwide. The roundworm (*Ascaris lumbricoides*), the whipworm (*Trichuris trichiura*), and hookworms (*Necator americanus* and *Ancylostoma duodenale*) are the major Soil-transmitted helminths (STHs) that infect humans. Unlike *T. trichiura* and *A. lumbricoides*, which are prevalent among young children, *N. americanus* and *A. duodenale* tend to infect older children and adults [4,5]. Soil-transmitted helminths are ubiquitous in tropical climates and rural temperate areas with inadequate sanitation facilities—that is, mostly poverty-stricken areas across the world. There is no vaccine for any human helminth infections, and current control efforts focus on mass drug administration, which is only partially effective [6]. Currently, the World Health Organization recommends that anthelmintic drugs such as mebendazole and albendazole are commonly used for mass administration to control STHIs [7], but they do not give lasting protection against re-infection. Meanwhile, other treatment drugs such as niclosamide (for *Taenia saginata*), piperazine (for *A. lumbricoides*), and ivermectin (for *Strongyloides stercoralis*) [8] are not suitable for mass control.

Eradication of helminths is challenging, mainly due to their complex life-cycles. Hookworms enter the human hosts when their infective stage 3 larvae (L3) penetrate the skin. Initially, L3 migrates through subcutaneous venules and lymphatics and then subsequently enters the afferent circulation to reach inside the lungs [4]. From there, L3 break through the alveolar spaces and are coughed up and swallowed into the gastrointestinal tract as they mature into L4 and adulthood. Maturation proceeds in the small intestine, where L3 moult twice to become adult male and female hookworms. After mating, female worms produce thousands of eggs that exit the body in the faeces. Whipworms, on the other hand, enter the human host through the ingestion of embryonated eggs from the environment. First-stage (L1) larvae hatch from the eggs and penetrate the epithelial cells at the crypt base to undertake an intracellular existence, where larvae grow and moult through the larval (L2, L3 and L4) and adult stages [9]. Examining helminth eggs in stool samples by Kato-Katz thick-smear technique is the only widely used diagnostic tool in helminth epidemiology [10,11], but this technique is less sensitive in the case of low-intensity helminth infections. Alternative sensitive diagnostic tools such as FLOTAC (a multivalent faecal egg count technique) and McMaster are available [7,12], but they are not adequate. It is, therefore, essential to understand these helminths holistically and devise precise diagnostic tools and effective treatment regimens that would provide long-term protection against diseases caused by STHs.

While there are numerous published studies on STH genomes [13,14,15,16,17] and proteomes [18,19,20], less attention has been afforded to their metabolomes [21,22] and, specifically, the lipidome [23]. A number of studies [24,25] have shown the importance of STH excretory/secretory products (ESPs) in host–parasite communication, including parasite survival and host protection against immunopathology. For example, ESPs such as TT47 and TM43 produced by whipworms are important for invasion of the gut wall by forming a pore in the epithelial cell membrane [26], and the major ESP, p43m, suppresses secretion of IL-13, a cytokine with anti-helminth properties [27]. Adult *Necator* and *Ancylostoma* hookworms release proteases [28] and anti-coagulant peptides [29] into the infection site to digest the host’s mucosal tissues and secrete abundant netrin-like proteins to suppress inflammatory responses by inducing regulatory T cells [30]. Moreover, a small-molecule linoleic acid, used by the cercariae of *Schistosoma mansoni* to produce prostaglandin, PGE2, helps them to successfully migrate inside their host [31]. However, very little is known about the small-molecule complement of STHs, particularly of their infective stages.

Since it is challenging to obtain parasite material from humans, in this study, we used the rodent model STH parasites, *N. brasiliensis* (model organism for the human hookworms *Ancylostoma* and *Necator* spp.) and *T. muris* (model organism for whipworm *T. trichiura*) to characterise and identify small-molecule components of their infective stages. Previously, we reported the ESP metabolomes of their adult developmental stages, both of which reside in the gut [21]. In this study, we hypothesised that the capacity of hookworms and whipworms to establish infection successfully might be related to the types of metabolites produced by their infective stages in the lung and gut, respectively. These findings would shed light on whether the metabolomes of infective-stage hookworms and whipworms are conserved or display stage-, species- or niche-specific molecules. Moreover, some of the metabolites discovered herein might be useful as potential diagnostic biomarkers.

## 2. Results

### 2.1. MSI Level-1 Identification of Polar Metabolites Present in the Infective Stages of N. brasiliensis and T. muris

**Figure 1:**
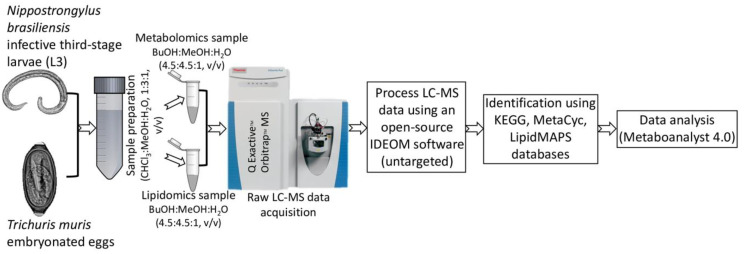
Schematic flowchart of the metabolomic and lipidomic profiling strategy applied for the infective stages of the *N. brasiliensis* (L3) and *T. muris* (embryonated eggs).

**Figure 2:**
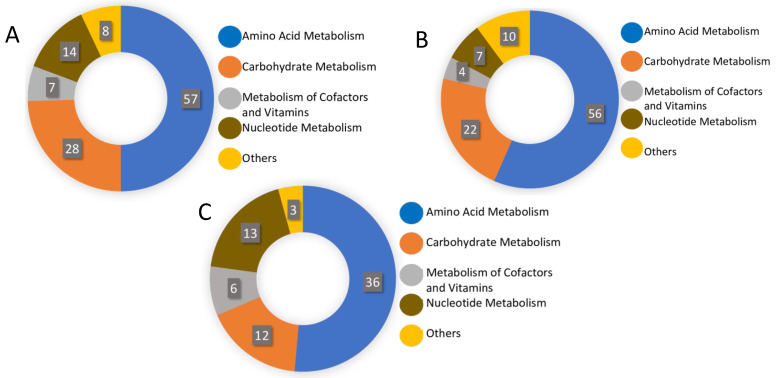
Distribution of total putative metabolites by different metabolite classes (**A**) in the somatic extract of infective third-stage larvae (L3) of *N. brasiliensis* (NB_SE); (**B**) in the excretory/secretory products of *N. brasiliensis* L3 (NB_ESP); and (**C**) in the somatic extract of *T. muris* embryonated eggs (TM_SE).

Using an untargeted LC-MS protocol, we analysed the metabolome composition of the infective stages of *N. brasiliensis* and *T. muris* as outlined in the Methods (Figure 1). From the infective L3 stage of *N. brasiliensis*, we prepared two biological samples—somatic tissue extract and ESP—and each biological sample had five replicates. The replicates of the L3 somatic tissue extract were named NB_SE_1 through NB_SE_5; ESP replicates were named NB_ESP_1 through NB_ESP_5. From the infective embryonated eggs of *T. muris*, we prepared five replicates of the somatic tissue extract, which were named TM_SE_1 through TM_SE_5. For the quality control, we prepared a pooled quality control from the samples (QC_P_1–QC_P_3) and media control (QC_Media_1–QC_Media_3) from the same media used for the experimental cultures. Using Metabolomics Standards Initiative level-2 (MSI-II) identification based on accurate mass and predicted retention time, using the open-source software IDEOM (an Excel interface for analysis of LC-MS-based metabolomic data), we identified a total of 164 putative polar metabolites altogether (i.e., after subtracting media peak area values from each metabolite) (Supplementary Table S1 from three different samples (NB_SE, NB_ESP and TM_SE). Most of the polar metabolites were the products of amino acid metabolism, followed by carbohydrate metabolism and nucleotide metabolism (Figure 2A–C).

Of these 164 putative polar metabolites, authentic chemical standards were available for 55 metabolites, allowing confident identification of 55 polar metabolites by accurate mass and retention time (MSI-I identification) (Table 1). Of the 55 metabolites, 21 were common to all three samples (NB_SE, NB_ESP, and TM_SE), comprising mostly products of amino acid metabolism, followed by carbohydrate and nucleotide metabolism. Based on the intensity of peak areas, adenosine, betaine, adenine, lactose, and choline were the top five compounds present in NB_SE. In NB_ESP, betaine, (S)-malate, l-glutamine, isocitrate, and 5-oxoproline were present as the major compounds. TM_SE contained l-leucine, lactose, adenosine, l-proline, and urocanate as the major metabolites (Table 1).

### 2.2. Metabolic Pathways of Putatively Identified Polar Metabolites

Based on the 164 putative polar metabolites identified from the three biological samples (114 metabolites from NB_SE, 99 from NB_ESP, 70 from TM_SE), we deduced metabolic pathways (MAPs) by mapping all the compounds against known metabolite pathways for helminths in the Kyoto Encyclopedia of Genes and Genomes (KEGG), Human Metabolome Database (HMDB), and MetaCyc Metabolic Pathway database. In all three sample groups, the highest number of identified metabolites/compounds were products of amino acid metabolism, followed by carbohydrate metabolism in NB_SE and NB_ESP, and nucleotide metabolism in TM_SE, as shown in Figure 2A–C. For example, of 114 metabolites identified from the NB_SE, 57 metabolites were produced through amino acid metabolism, 28 by carbohydrate metabolism, and 14 by nucleotide metabolism. A similar pattern can be seen in its ESP. Of 70 polar metabolites identified from TM_SE, 36 of them were the products of amino acid metabolism, 13 were from nucleotide metabolism, and 12 were from carbohydrate metabolism.

### 2.3. Chemometric Analysis of the Polar Metabolites of N. brasiliensis L3 and T. muris Embryonated Eggs

**Figure 3:**
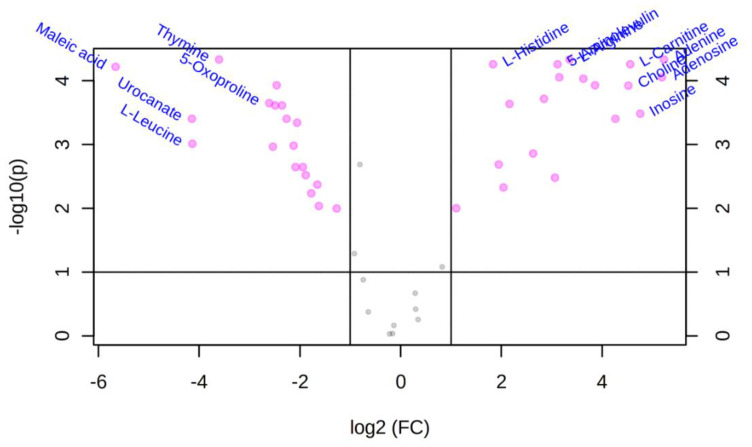
Volcano plot of untargeted metabolomics analysis of differential features (i.e., 49 MSI-I (Metabolomics Standard Initiative level-1) identified polar metabolites from somatic extracts) between the somatic extract of infective third-stage larvae (L3) of *N. brasiliensis* (NB_SE) and the somatic extract of *T. muris* embryonated eggs (TM_SE). The volcano plot displays log2 fold changes versus Benjamini–Hochberg adjusted *p*-values (−log10 transformed). Features that exhibited an absolute log2 fold change > 2 and an absolute *p*-value < 0.05 are coloured in pink. Most significant metabolites are labelled with the corresponding name.

We performed statistical analysis on the 55 polar metabolites identified in Table 1 (MSI-I identification) using MetaboAnalysis 4 (http://www.metaboanalyst.ca) to determine metabolite differences. First, the differences in the abundance of polar metabolites between NB_SE and TM_SE were evaluated by univariate analysis (volcano plot analysis). NB_ESP was excluded from the univariate analysis since there was no ESP from the infective stage of *T. muris*. Volcano plot analysis identified differential metabolites using the *t*-test and fold-change (FC) methods, and we plotted log_2_ (fold-change > 2) on the *X*-axis against *-log*_10_ (*p*-value) from the *t*-test on the *Y*-axis. Benjamini–Hochberg correction or false discovery rate was applied to compute the number of false positives out of significantly varied metabolic features. Table 1 shows the fold change and the *p-*values of these significantly different metabolites. When we compared NB_SE metabolites against TM_SE, 38 of 49 (approximately 78%) metabolites showed significant differences (*p* < 0.05), where both the samples (i.e., NB_SE and TM_SE) had 19 metabolites each with an absolute log2 fold change > 2 and an absolute *p*-value < 0.05 (represented as pink dots in Figure 3).

**Figure 4:**
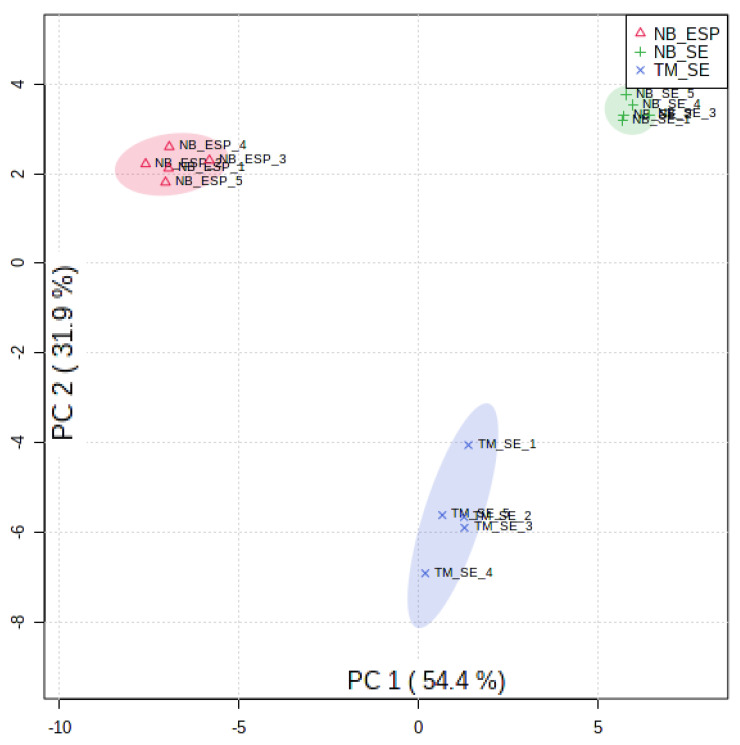
Principal component analysis scores plot for three sample groups, i.e., the somatic extract of infective third-stage larvae (L3) of *N. brasiliensis* (NB_SE) (green), the excretory/secretory products (ESP) of *N. brasiliensis* L3 (NB_ESP) (red), and the somatic extract of *T. muris* embryonated eggs (TM_SE) (blue) with five replicates each. Parentheses on each axis explain the amount of variance. Media (QC_Media) and pooled quality control (QC_P) values were excluded from the analysis, as they were subtracted from the samples.

We next performed multivariate analysis by a principal component analysis (PCA) and hierarchical clustering analyses (with heat-map analyses) to determine the overall metabolic similarities and differences between three sample groups (i.e., NB_SE, NB_ESP, and TM_SE). As expected, the PCA plot (Figure 4) showed a clear separation among the three biological groups, indicating that the infective stages of the two STHs (*N. brasiliensis* and *T. muris*) produce distinct metabolite profiles. The plot shows that the variation of metabolites between the different helminth species and the difference shown on PC1 was greater than the difference between somatic extract and excretory/secretory product.

**Figure 5:**
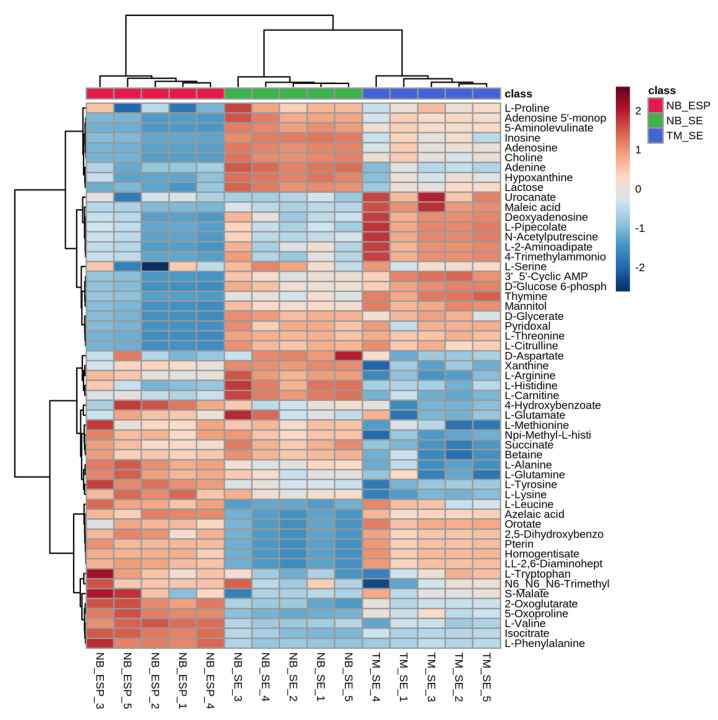
Hierarchical clustering analysis (HCA) of the three samples (the somatic extract of infective third-stage larvae (L3) of *N. brasiliensis* (NB_SE), the excretory/secretory products (ESP) of *N. brasiliensis* L3 (NB_ESP), and the somatic extract of *T. muris* embryonated eggs (TM_SE)) showing discrimination between the sample types and differential abundances of 55 polar metabolites. Each column represents sample groups with their replicates, and each row represents the expression profile of a metabolite across sample groups. The scale bar represents the normalised intensity of metabolites, where blue indicates a decrease/low and red an increase/high. Media (QC_Media) and pooled quality control (QC_P) values were excluded from the analysis, as they were subtracted from the samples.

Additionally, we used hierarchical clustering heat-map analysis (distance measured by Euclidean, and clustering algorithm using ward.D) to evaluate the difference in the concentration of each metabolite between sample groups. Clustered heat-maps allow easy visualisation of changing patterns in metabolite concentrations across sample groups and experimental conditions. The metabolite pattern of each biological replicate from three sample groups clustered, and individual clusters, were distinct, indicating the uniqueness of the metabolome profiles of the two helminths. Unlike score plots, heat-maps display the actual data values using carefully chosen colour gradients, as shown in Figure 5, where blue bars indicate a low concentration and red bar denotes a high concentration. Based on the colour intensity pattern in the heat-map, adenine (purine nucleobase), adenosine (purine nucleoside), choline (amino acid), and hypoxanthine were notable features in the NB_SE. Amino acids such as l-tyrosine, l-valine, 2-oxoglutarate, l-phenylalanine, and 4-hydroxybenzoate were prominent in NB_ESP. In TM_SE, l-2-aminoadepate, l-pipecolate, and N-acetylputrescine (amino acids), thymine (nucleic acid), and maleic acid (organic acid) were prominent.

### 2.4. Lipidomics Analysis of the N. brasiliensis and T. muris Infective Stages

After acquiring LC-MS data (mass and retention time) using open source software IDEOM, lipids were putatively identified by accurate mass within 3 ppm. We putatively identified (MSI level-2 identification) a total of 350 lipids (Supplementary Table S2) in all three samples (332 in NB_SE, 256 in NB_ESP, and 283 in TM_SE), out of which 203 lipids were common in all three sample groups. Of the vast array of lipids identified, glycerophospholipids such as phosphatidylcholine (PC) and phosphatidylethanolamine (PE) were predominant in NB_SE. In NB_ESP and TM_SE, glycerolipid triglycerides (TG) were present in higher intensities. Based on their intensity of peak areas, glycerophospholipid species phosphatidylcholine (PC) such as PC(38:6), PC(40:5), PC(40:7), PC(40:8), and PC(40:9) were the top five lipids in NB_SE. In NB_ESP and TM_SE, glycerolipid species such as triglyceride (TG) were dominant. TG(48:1), TG(45:0), TG(50:2), TG(47:1), and TG(52:2) were the top five lipids in NB_ESP and TG(30:0), TG(38:0), TG(38:1), TG(40:0), and TG(42:1) in TM_SE.

### 2.5. Lipidomic Pathways of Identified Lipids

**Figure 6:**
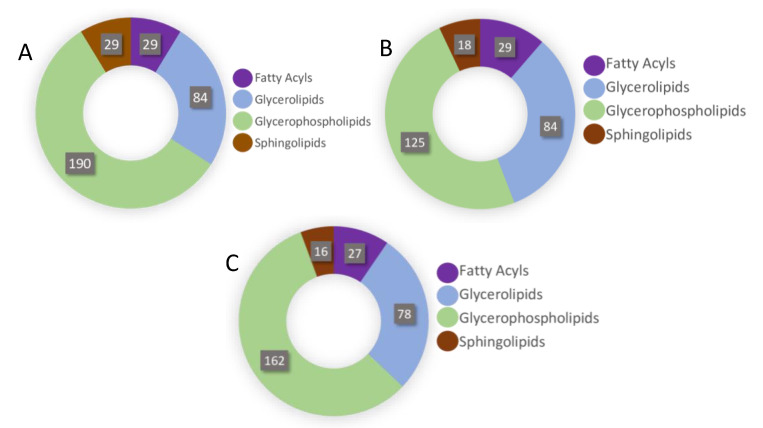
Distribution of putative lipids by lipid classes: (**A**) in the somatic extract of infective third-stage larvae (L3) of *N. brasiliensis* (NB_SE); (**B**) in the excretory/secretory products (ESP) of *N. brasiliensis* L3 (NB_ESP); (**C**) in the somatic extract of *T. muris* embryonated eggs (TM_SE).

We mapped lipidomic pathways for putative lipids against known lipid pathways in lipid databases such as LIPID MAPS Lipidomics Gateway (http://www.lipidmaps.org) and LipidBank (http://www.lipidbank.jp). Most lipids in all three sample groups were the glycerophospholipid metabolism pathway products, followed by glycerolipid and fatty acyl metabolism (Figure 6). Elements of glycerophospholipid metabolism such as phosphatidic acid (PA), phosphatidylethanolamine (PE), phosphatidylcholine (PC), phosphatidylserine (PS), and phosphatidylinositol (PI) were major lipid constituents in all three sample groups (NB_SE, NB_ESP, and TM_SE), followed by triacyl-glycerides (TG), the product of the glycerolipid pathway (Supplementary Table S2).

### 2.6. Chemometric Analysis of Putatively Identified Lipids

**Figure 7:**
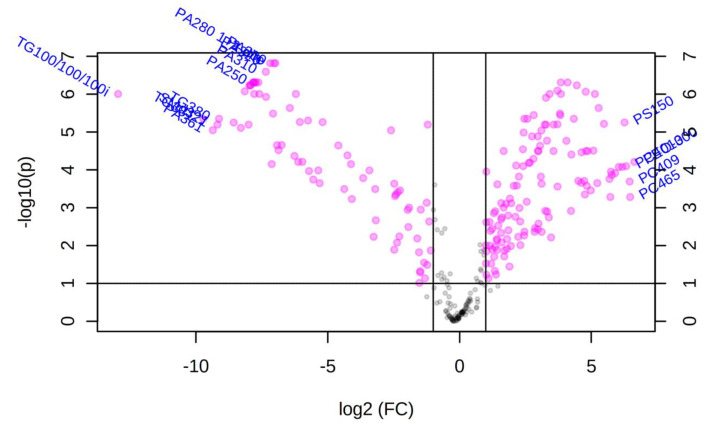
Volcano plot of the untargeted lipidomics analysis of differentially regulated features between the somatic extract of infective third-stage larvae (L3) of *N. brasiliensis* (NB_SE) and the somatic extract of *T. muris* eggs (TM_SE). The volcano plot displays log2 fold changes versus Benjamini–Hochberg adjusted *p*-values (-log10 transformed). Features that exhibited an absolute log_2_ fold change > 2 and an absolute *p*-value < 0.05 are coloured in pink. Most significant features which could be structurally annotated are labelled with the corresponding name. Gray dots represent features that were not significantly altered (having fold change less than 2). The further its position away from the (0,0), the more significant the lipid is.

We performed both univariate and multivariate chemometric analyses using Metaboanalyst 4.0 software, as described above for polar metabolites. For comparison of lipid profiles in the somatic extracts of the infective stages of the two helminths, univariate analysis (volcano plot analysis) between NB_SE and TM_SE yielded 204 significant features out of top 350 lipids (represented by pink dots in Figure 7). All features presented possessed values above a given count threshold (>75% of pairs/variable) and had a fold change of >2 and *p*-value of <0.05 (Supplementary Table S3). When the NB_SE lipid profile was compared against TM_SE, 10 lipids in NB_SE and five lipids in TM_SE (labelled pink dots in Figure 7) showed the most significant differences in their peak intensities (*p* < 0.05).

**Figure 8:**
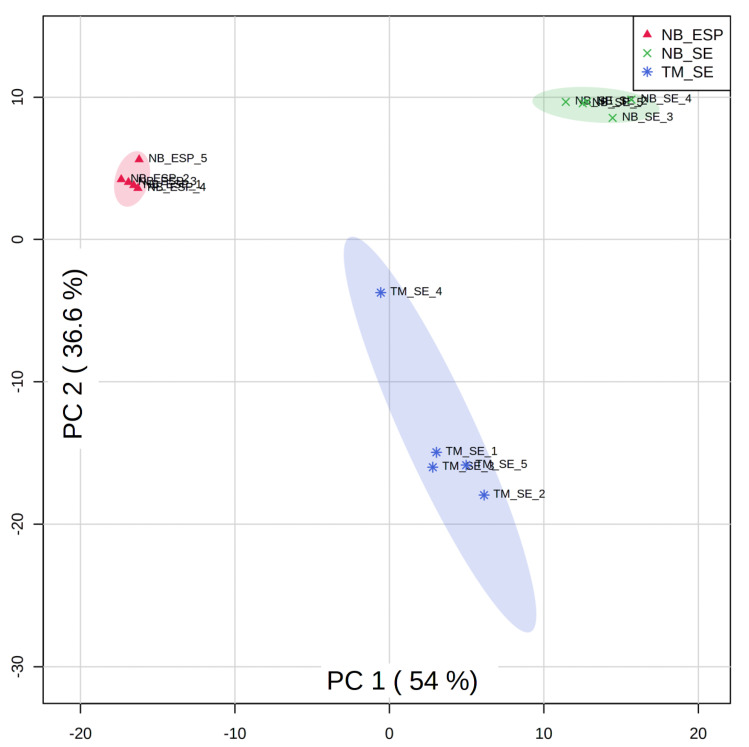
Principal component analysis scores plot comparing LC-MS metabolomic profiles for the lipids among three sample groups: the somatic extract of infective third-stage larvae (L3) of *N. brasiliensis* (NB_SE) (green), the excretory/secretory products of *N. brasiliensis* L3 (NB_ESP) (red), and the somatic extract of *T. muris* embryonated eggs (TM_SE) (blue) with five replicates each. The amount of variance explained is shown in parentheses on each axis. Media (QC_Media) and pooled quality control (QC_P) values were excluded from the analysis, as they were subtracted from the samples.

A principal component analysis of the 350 putative lipids (listed in Supplementary Table S2) showed clear segregation of the clusters among all three sample groups (NB_SE, NB_ESP, and TM_SE) as presented in Figure 8. These results indicate a high level of heterogeneity in the lipidomes of the two helminths.

**Figure 9:**
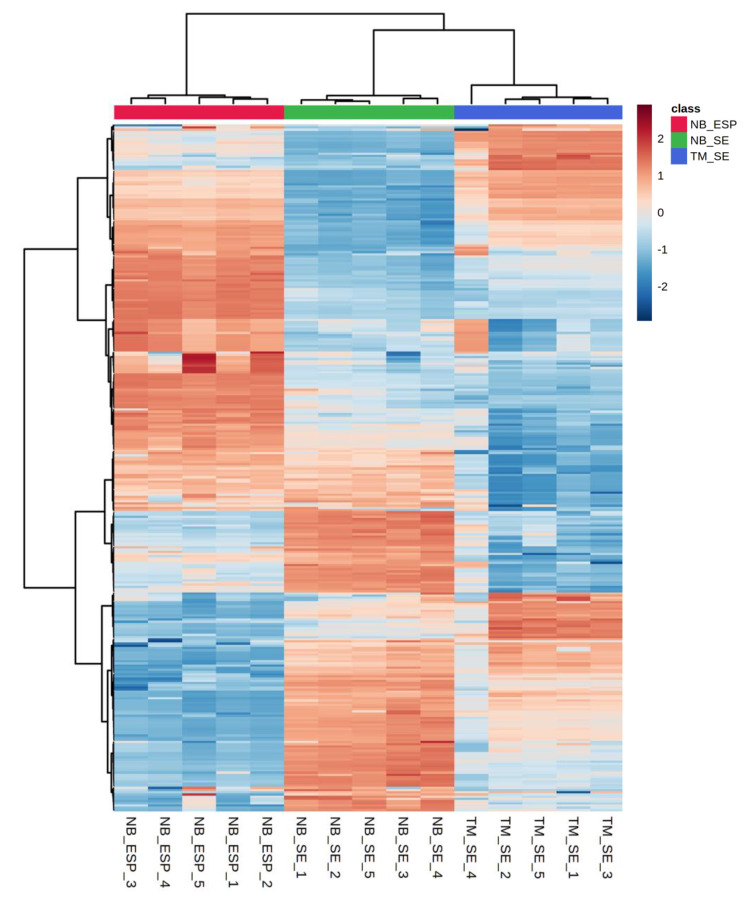
Hierarchical clustering analysis (HCA) of the three samples (the somatic extract of infective third-stage larvae (L3) of *N. brasiliensis* (NB_SE), the excretory/secretory products (ESP) of *N. brasiliensis* L3 (NB_ESP), and the somatic extract of *T. muris* embryonated eggs (TM_SE)), showing discrimination between the sample types and differential abundances of 350 putative lipids. Each row represents putative lipids, and each column represents the biological replicates of an individual sample group. The scale bar represents the normalised intensity of metabolites, where blue colour indicates low/decrease, and red colour indicates high/increase. Media (QC_Media) and pooled quality control (QC_P) values were excluded from the analysis, as they were subtracted from the samples. Note: Heat-map was purposefully generated on all 350 putative lipids to show the overall pattern between the sample groups. For specific lipid details with peak areas, refer to Supplementary Table S2.

We evaluated the diversity of lipidomic patterns across the three samples by cluster heat-map analysis using Metaboanalyst 4.0 statistical analysis package. The variation in the intensities of 350 putative lipids between groups is shown in Figure 9. The somatic extract of *N. brasiliensis* L3 contained a very high intensity of glycerophospholipids such as phosphatidylcholine (PC) and phosphatidylethanolamine (PE) species. Meanwhile, NB_ESP and TM_SE both contained glycerolipids such as triglycerides (TG) species in higher intensities. The Supplementary Table S2 contains the individual intensity of all 350 lipids in each group.

### 2.7. Common and Unique Metabolites in N. brasiliensis and T. muris Infective Stages

**Figure 10:**
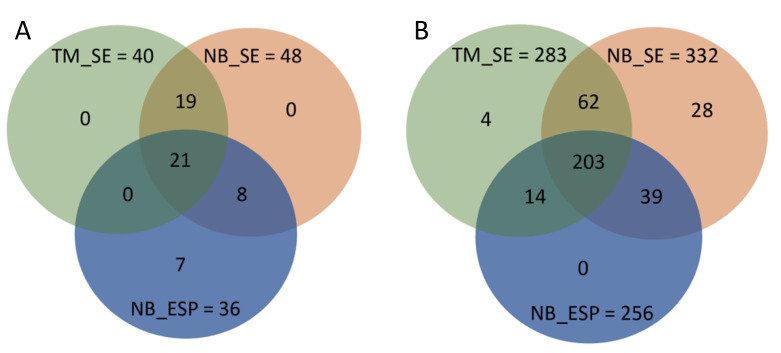
Distribution of common and unique metabolites among the three sample groups: the somatic extract of infective third-stage larvae (L3) of *N. brasiliensis* (NB_SE), the excretory/secretory products (ESP) of *N. brasiliensis* L3 (NB_ESP), and the somatic extract of *T. muris* embryonated eggs (TM_SE). (**A**) MSI-I identified polar metabolites; (**B**) MSI-II identified lipids.

Out of 55 polar metabolites identified in this study, 21 were common to all three sample groups, mostly amino acids. Seven (7) polar metabolites were unique to the ESP of the *N. brasiliensis* infective stage (Table 1 and Figure 10A), and interestingly, two of them, namely isocitrate and 5-oxoproline/pyroglutamic acid, were also detected in their adult ESP [21]. The remaining five, namely homogentisate, orotate, ll-2,6-Diaminoheptanedioate, pterin, and 2,5-dihydroxybenzoate, were absent in the ESP as well as somatic extracts of both helminths. Meanwhile, out of 350 putative lipids identified from all three samples (NB_SE, NB_ESP, and TM_SE), 203 lipids were common to all three sample groups (NB_SE, NB_ESP, and TM_SE). A total of 62 lipids were common between NB_SE and TM_SE, 39 lipids between NB_SE and NB_ESP, and 14 lipids between NB_ESP and TM_SE. Twenty-eight (28) lipids were unique to NB_SE and four lipids to TM_SE (Table 2 and Figure 10B).

### 2.8. Reported Pharmacological Activities of Identified Compounds

A comprehensive literature search revealed that 42 out of the 55 polar metabolites were associated with various pharmacological activities. Of the 42 polar metabolites, 31 metabolites possess anti-inflammatory or antioxidant properties (Table 1). For example, polar metabolites such as l-valine (prominent in *N. brasiliensis* ESP), adenine, choline, inosine, adenosine, hypoxanthine (prominent features in *N. brasiliensis* somatic extract), and l-tryptophan (prominent feature in *T. muris* eggs somatic extract) possess anti-inflammatory activity. A few of the detected metabolites were unique to the extracts, such as homogentisate in the *N. brasiliensis* ESP and maleic acid in *T. muris* eggs (embryonated) somatic extract, both (compounds) known for their inflammatory and cytotoxic properties, respectively. Unlike polar metabolites, non-polar metabolites or lipids in helminths are the least studied for biological activities. There is either limited or no record of studies for all putative lipids identified in this study.

## 3. Discussion

Parasites have co-evolved with humans for millennia and produce ESPs that allow them to navigate through circuitous pathways (for hookworm at least) to reach the gut, where they survive for a prolonged period. STHs are masterful at modulating the host’s immune response to avoid elimination from the body and facilitate the establishment of chronic infection [92]. Their ESP contain a plethora of biomolecules, including proteins, peptides, lipids, and other small molecules [21]. However, small molecules remain less-studied, especially those produced by the infective stages. In this study, we show the metabolomes of the infective stages of *N. brasiliensis* and *T. muris* using untargeted LC-MS for the first time. Our results also provide new insights into comparative metabolome profiles of different developmental stages of the helminths. LC-MS is a preferred analytical technique for metabolome profiling [93,94,95], especially when employing high-resolution accurate mass (HRAM) detection. In this study, we applied HRAM mass spectrometer-Q-Exactive (Thermo Scientific) to detect both polar metabolites and lipids.

Helminths are known to produce stage-specific metabolites [21]. Although both adult and infective stages of *N. brasiliensis* had many fatty acids in common, interestingly, 44 of the polar metabolites detected in the infective stage of *N. brasiliensis* were not detected in the ESP of their adult stage [21]. Such differences in the metabolic profiles could have resulted partially due to the different experimental conditions and analytical techniques used in two separate studies. Nevertheless, such marked differences in the metabolic profiles of two different life-cycle stages of *N. brasiliensis* suggest that there could be major metabolic changes accompanying the transition from one life-cycle stage to the next in parasitic helminths. Moreover, according to Barrett [96], there are variations in the distribution pattern and activity of enzymes and metabolite levels among the different developmental stages of helminths. For instance, adult and larval stages of *A. lumbricoides* and *S. mansoni* possess a marked difference in the isoenzyme patterns. When we compared metabolites identified from the somatic extracts of *N. brasiliensis* and *T.* *muris* infective stages, the percentage similarity for both polar and non-polar metabolites was quite low (34.5% and 18%, respectively) despite sharing some common metabolites such as l-citrulline, l-threonine, deoxyadenosine, and pyridoxal. Metabolites such as inosine, hypoxanthine, and l-pipecolate were comparatively higher in NB_SE and were also reported significantly in high levels in the blood plasma samples of patients suffering from onchocerciasis (caused by nematode *Onchocerca volvulus*) [97]. The lower percentage similarity of metabolites in the somatic extracts of the two helminths could have resulted from the different conditions they were exposed to, as *N. brasiliensis* L3 were incubated at 26 °C with activated charcoal and later at 37 °C, 5% CO_2_ in glutamax/Phosphate-buffered saline (PBS) media. At the same time, *T. muris* eggs were kept in PBS at room temperature. Thus, suggesting that the different environmental niches in which they survive—for instance, the L3 of human hookworms *N. americanus* and *A. duodenale* remain outdoors, while their adult stage dwells inside the host gut [4]—could potentially influence the types as well as the level of metabolites produced. However, the effects of CO_2_ tensions and temperature on the overall metabolic pathways are either complex or difficult to predict [96].

More than half of the metabolites identified in all sample groups were amino acids. l-citrulline, l-methionine, N(pi)-Methyl-l-histidine, succinate, and 4-hydroxybenzoate were the top five most abundant metabolites (based on LC-MS peak intensity) in the somatic extract and excretory/secretory products of *N. brasiliensis* L3. Succinate, one of the amino acids detected in the current study samples, is known to be produced by the intestinal microbiota to induce intestinal tuft cells to trigger T-helper cell type 2 (T_H_2) responses [98]. Somatic and ESP of the adult stage of *N. brasiliensis* also contained succinate [21]. In the *T. muris* embryonated eggs, l-2-aminoadepate, N-acetylputrescine, l-pipecolate, thymine, and maleic acid were present in the highest intensities. Betaine is another amino acid detected in the infective stage of *N. brasiliensis* but not in their adult stage [21]. Betaine (a derivative of amino acid glycine with three methyl groups), present in microorganisms, plants and animals, is known to function as an osmolyte in their cells [99]. The plasma and spleen of rats infected with the liver fluke *Fasciola hepatica* also contained a high level of betaine [100]. Another metabolite that was prominent in both *N. brasiliensis* L3 ESP and *T. muris* embryonated eggs was l-glutamate. The dormant infective eggs of other helminths such as *A. lumbricoides* also contained l-glutamate [101]. The relevance of betaine and l-glutamate (and glutamine) in this study should be interpreted with caution, as it is possible that samples were contaminated by the sulfobetaine polymer (PSB) used for the somatic extract preparation and the glutamax used in the media. Pyroglutamic acid (also called 5-oxoproline), reported as one of the major metabolites in the somatic extracts of the adult stage of *N. brasiliensis* and *T. muris* [21], was a common metabolite in the infective stages of two helminths. Somatic extract of adult *Ancylostoma caninum* (dog hookworm) also contained pyroglutamic acid as one of the major metabolite constituents [102]. Pyroglutamic acid is formed as an intermediate product by the enzyme γ-glutamylcyclotransferase in glutathione metabolism, and it is ultimately converted into l-glutamic acid by 5-oxoprolinase [103]. It is also known to be produced due to disordered glutathione metabolism [104] and usually tends to accumulate abnormally in the case of metabolic acidosis.

Another exciting difference among the samples from two helminths is the presence of unique metabolites. Seven polar metabolites, namely orotate, pterin, 2,5-dihydroxybenzoate, ll-2,6-Diaminoheptanedioate, isocitrate, 5-oxoproline, and homogentisate, were found unique to the ESP of *N. brasiliensis*. Out of these seven, only isocitrate and 5-oxoproline were reported in their adult ESP [21]. In mammals, orotate (orotic acid) is released by mitochondrial dihydroorotate dehydrogenase for conversion to uridine monophosphate during pyrimidine metabolism [105]. Moreover, five main enzymes involved in pyrimidine metabolism are present in many helminths, including *N. brasiliensis* and *T. muris* [106], indicating that de novo pyrimidine biosynthesis could be the main source of orotate in helminths. Pterins are found in all living organisms starting from tiny bacteria to mammals and serve as a urine biomarker for hyperphenylalaninaemia [107]. Homogentisate is the central intermediate product in the catabolism of phenylalanine and tyrosine [108]. Whilst we have not ruled out the possibility that this feature could be another isomer of dihydroxyphenylacetate, it is most likely an intermediate in tyrosine/phenylalanine metabolism. Tyrosine catabolism is considered as a critical metabolic pathway in *Rhodnius prolixus,* a blood-sucking insect vector of *Trypanosoma cruzi*, because *R. prolixus* dies after a blood meal if the pathway is disturbed by silencing two critical enzymes, tyrosine aminotransferase and 4-hydroxyphenylpyruvate dioxygenase [109]. Thus, the presence of phenylalanine and tyrosine in *N. brasiliensis* L3 ESP could mean that tyrosine catabolism may be important for the survival of *N. brasiliensis* L3 when establishing a successful infection. Homogentisate also possesses antioxidant activity higher than α-tocopherol and moderate anti-inflammatory activity [110]. Thus, the capacity of ESP from many helminths, including *N. brasiliensis*, to reduce T-cell proliferation [111] and confer protection against T-cell mediated immunopathology in a mouse model of colitis [102], are attributable to the presence of such metabolites.

Identifying different metabolic pathways within a species is considered necessary, mainly to understand any malfunctions or alterations that may occur during disease state [112]. Similarly, identifying metabolic pathways in soil-transmitted helminths might reveal a unique metabolic pathway(s) critical in the infection process and, therefore, present as drug targets. The metabolic pathway analysis revealed that the majority of the metabolic pathways are associated with amino acid metabolism, a finding that aligns with earlier metabolic profiling of the ESP from adult stage STHs [21], suggesting that both the infective and adult stages share similar (amino acid and carbohydrate) metabolic pathways. Polar metabolites identified from the infective stages of both helminths mostly belonged to common amino acid pathways such as aminoacyl-tRNA biosynthesis, arginine biosynthesis, lysine degradation, alanine, aspartate, and glutamate metabolism. Amino acids, such as leucine, lysine, and phenylalanine, are detected significantly in high concentrations in the herbivorous youngstock acutely infected with helminths [113]. l-arginine is known to enhance intestinal mucosa function by reducing tissue damage in intestinal ischemia of animal models [114,115]. We also found purine, glyoxylate, and dicarboxylate metabolism as major pathways following amino acid pathways. Glyoxylate metabolism, which is characteristic of free-living parasitic nematodes (but not in the adult) and catalyses the conversion of lipids into carbohydrates [96], could be the source of isocitrate (organic acid), which was one of the unique metabolites in the *N. brasiliensis* ESP.

Lipids and fatty acids are also known to play a crucial role in the maturation and completion of different life-cycle stages of helminths and host–parasite interaction [23]. Generally, the parasitic stage of helminths utilise lipids for the long-term adaptation inside their host and completion of the life-cycle [116]. Lipids are also involved in essential biological processes such as apoptosis, cell proliferation, angiogenesis, immunity, and inflammation [117]. Fatty acids, including the *cis-*form of octadecenoic acid and other branched-chain and monoenoic acids (oleic acids and vaccenic acid), are known to play a vital role in helminth infections by altering the physical properties of the host cell membrane and ultimately causing it to rupture [118]. Thus, we presume that fatty acids, such as octadecanoic acid detected in the ESP of *N. brasiliensis* L3, might be playing a key role during the process of host invasion and infection. Fatty acids were also reported in other nematodes such as *Haemonchus contortus* (in all stages of life-cycle) [119] and adult *Caenorhabditis elegans* [120], and in both studies, fatty acids with 18 carbons (i.e., 18:1, 18:2, and 18:3) were commonest. We also obtained a similar result, where ~7% of total lipids were fatty acids, and the above-mentioned fatty acids with 18 carbons were present in all three samples (i.e., NB_SE, NB_ESP, and TM_SE). Nematodes regulate the saturation levels of fatty acids while adapting to the changing environmental temperature [121], but the saturation level varies among different nematodes. For example, in *C. elegans*, the saturated fatty acid level increases with increasing temperature [122]. In contrast, Wang et al. [119] observed the opposite in *H. contortus*, where the level of fatty acid saturation tended to decrease as they transitioned from their free-living stage to the parasitic stage.

Glycerophospholipids and glycerolipids were the major lipid groups in all three sample groups (NB_SE, NB_ESP, and TM_SE), constituting approximately 83% of the total lipids. This is in congruence with lipidomic studies in the muscle-stage larvae of *Trichinella papuae* (~63% glycerophospholipids) [123] and in the *H. contortus* (all life-cycle stages), where more than 90% of the total lipids were glycerophospholipids and glycerolipids [119]. The somatic extract of *N. brasiliensis* L3 contained elements of glycerophospholipids such as phosphatidylcholine, PC(38:6), PC(40:8), PC(40:7), PC(40:9), and PC(46:5). Wewer et al. [124] also reported PC as dominant lipid constituents in filarial nematodes *Onchocerca volvulus*, *O. ochengi,* and *Litomosoides sigmodontis* but they have studied only their adult stage. Meanwhile, glycerolpids such as TG(45:0), TG(48:1), TG(50:2), TG(30:0), TG(38:0), and TG(38:1) were dominant in the excretory/secretory product of *N. brasiliensis* L3 and somatic extract of *T. muris* embryonated eggs. TG constituted major lipids (80.9% of total 327 lipids) identified from the somatic extract of the L3 stage of *H. contortus* and it decreases as they mature into L4 stage [119]. Lee [125] also reported triglycerides as a major lipid constituent in the adult tissue of *N. brasiliensis*, which was presumed to be due to reduced lipid metabolism under anaerobic conditions inside their host. Triglyceride is the major neutral lipid in the majority of helminths [126]. According to Ward [118], unlike mammals, helminths are capable of storing a large amount of energy in the form of glycerolipids as triglycerides or triacylglycerides have more energy content (9 kcal/g) compared to carbohydrates (4 kcal/g). Thus, it is likely that infective stages of both helminths *N. brasiliensis* L3 and *T. muris* embryonated eggs store energy in the form of triglycerides for their later developmental stages of the life-cycle. Our findings complement a similar study where the *Schistosoma mansoni* infective stage (cercariae) had a unique lipid profile compared to other stages of the life-cycle [23]. Interestingly, although *S. mansoni* belongs to a different phylum (Platyhelminthes), it shares some behavioural aspects with *N. brasiliensis*—notably, the penetration of host skin by the infective stage larvae. Although we could not retrieve literature on pharmacological activities of specific triglycerides identified in this study, a high level of triglycerides was associated with inflammation and inflammation-related disorders [127]. *T. muris* embryonated eggs showed enrichment of species of phosphatidic acids (PA), out of which PA(25:0) and PA(26:0) were completely absent in the infective stage L3 of *N. brasiliensis*. Phosphatidic acid is a lipid of interest as a vital signalling molecule and a central intermediate in the synthesis of membrane lipids and storage lipids [128,129]. Phosphatidic acid, such as lysoPA (C14:2), was also detected in the surface coat of the infective stage of the parasitic nematode *Trichinella spiralis* [130]. Other species of helminths such as *Hymenolepis diminuta*, *A. lumbricoides*, *Dirofilaria immitis*, and *Setaria cervi* also contained phosphatidic acids [126]. Phosphatidylcholines (PC) reported to be produced by all life-cycle stages of parasitic trematode [23] were also detected in both helminths studied here. PC lipids are associated with the maintenance of gastrointestinal mucus barrier function [131], besides their good anti-inflammatory properties [132,133]. PC lipids such as PC(36:7), PC(P-32:2), and PC(P-36:2) were unique to *N. brasiliensis* L3 somatic extract.

## 4. Materials and Methods

### 4.1. Ethics, Source, and Housing of Mice and Rats

Mice strain B10.Br (5 weeks old, 5 mice per cage) and rat strain Sprague–Dawley (2 weeks old, 2 rats per cage) were purchased from Animal Resources Centre (Perth, Australia). All experiments using these animals were approved by the animal ethics committee of James Cook University (JCU), Cairns, Australia (animal ethics number: A2647). Mice and rats were kept in the JCU animal facility centre in compliance with JCU approved protocols, Australian Code of Practice for the Care and Use of Animals for Scientific Purposes, (7th edition, 2007), and the Queensland Animal Care and Protection Act 2001. The animal facility room had an ambient temperature (20–22 °C) and humidity (60%), and animals were exposed to a 12 h day/night cycle and fed irradiated mouse/rat chow (Specialty Feeds, Glen Forrest, Western Australia) and autoclaved tap water ad libitum.

### 4.2. Collection of N. brasiliensis L3 and Its ESP

Sprague–Dawley rats were infected with *N. brasiliensis* L3 (~3000 larvae) by subcutaneous injection and sacrificed on day seven post-infection [134]. We collected faecal pellets on day five and six post-infection. Subsequently, faecal pellets were cultured with activated charcoal—untreated, granular, 8–20 mesh (Sigma-Aldrich, New South Wales, Australia). The culture plates were sealed inside an airtight plastic container in an incubator (Binder, model: BD 115 #02-040007) at 26 °C for one week. After one-week incubation, L3 were harvested, washed with warmed 5× pen/strep PBS, and then transferred to a 12-well culture plate (1500 worms per well) containing warmed 2 mL 5× glutamax, 2× pen/strep PBS media. Plates were incubated in a CO_2_ incubator (Sanyo MCO-18AIC CO_2_ incubator, SANYO Electric Co., Ltd., Moriguchi, Japan) at 37 °C supplied with 5% CO_2_. The supernatant (ESP) was collected and replaced with fresh media twice daily (09:00 and 17:00) for four consecutive days. The supernatants were centrifuged at 3000× *g* for 30 min, and then aliquot was transferred to Amicon^®^ Ultra-15, centrifugal 10 kDa filters (Merck Millipore, Victoria, Australia), and centrifuged at 4000× *g* for 20 min. Concentrated ESP filtrate containing small molecules (<10 kDa) was collected and stored at −80 °C until further analysis.

### 4.3. Collection of Eggs (Infective Stage) from T. muris Adult Worms

B10.Br mice were infected with *T. muris* embryonated eggs (200 μL of PBS containing ~200 eggs) through oral gavage. We euthanised mice with CO_2_ on day 30–33 post-infection to harvest adult *T. muris* for egg collection. Caecum was collected, split longitudinally, and washed in warmed 5× pen/strep in PBS. Adult worms were carefully pulled from the caecum of mice with fine forceps and washed with 5x pen/strep in PBS, and then transferred to 6-well culture plates (~100 worms per well) containing 4 mL of warmed 5× glutamax, 2× pen/strep in PBS media. Worms were incubated in a CO_2_ incubator at 37 °C in 5% CO_2_. For egg collection, we replaced culture media with fresh media twice daily as described above for three consecutive days. The supernatant was collected twice daily and centrifuged at 3000× *g* for 30 min. Eggs from adult *T. muris* worms were resuspended in 40 mL milli-Q water and filtered through a 100 μm nylon sieve before transferring to a fresh cell culture tube. Finally, for eggs to become embryonated, eggs were kept in the sterile PBS and stored in the dark at room temperature for approximately six weeks, and then stored at 4 °C.

### 4.4. Somatic Extract Preparation

Somatic extracts of *N. brasiliensis* L3 (five biological replicates named as NB_SE_1, NB_SE_2, NB_SE_3, NB_SE_4, and NB_SE_5, each replicate containing ~17000 L3) and *T. muris* embryonated eggs (five biological replicates named as TM_SE_1, TM_SE_2, TM_SE_3, TM_SE_4, and TM_SE_5, each replicate containing ~17000 eggs) were suspended in 1 mL chilled sulfobetaine polymer (PSB) and centrifuged at 1000× *g* for 5 min at 4 °C. The supernatant was discarded, and the remaining solid was resuspended in 250 µL of chilled extraction solvent (CHCl_3_:MeOH:H_2_O, 1:3:1, *v*/*v*) containing 1 µM of internal standards 3-(cyclohexyl amino)-1-propane sulfonic acid (CAPS), 3-[(3-cholamidopropyl) dimethylammonio]-1-propanesulphonate (CHAPS), piperazine-N,N’-bis(2-ethane sulfonic acid (PIPES), and Tris(hydroxylmethyl)amino-methane (Tris). A blank sample containing water instead of a tissue pellet was extracted simultaneously as a control. After three freeze-thaw cycles, the samples were mixed thoroughly for 30 min at 4 °C. The samples were centrifuged at 14,800× *g* for 10 min at 4 °C. Supernatant (100 µL) was transferred to the vials for metabolomics analysis and analysed on the same day. Another 100 µL was transferred to microfuge tubes for lipidomics analysis. The solvent was evaporated using a centrifugal evaporator at 55 °C for 50 min. Dried extracts were frozen at −80 °C until LC-MS analysis was performed. On the day of analysis, the samples were dissolved in 80 µL of BuOH:MeOH:H_2_O (4.5:4.5:1, *v*/*v*). The samples were shaken for 30 min at room temperature and sonicated for 1 h while maintaining the temperature below 25 °C. The samples were centrifuged at 14,800× *g* for 10 min at 20 °C, and then 70 µL was transferred to LC-MS vials.

### 4.5. ESP Extract Preparation

ESP from *N. brasiliensis* L3 was thawed on ice and 100 µL aliquots of ESP (five biological replicates named as NB_ESP_1, NB_ESP_2, NB_ESP_3, NB_ESP_4, and NB_ESP_5) were transferred to microfuge tubes kept on ice. Subsequently, 400 µL of extraction solvent (CHCl_3_:MeOH, 1:3, *v*/*v*) containing 1 µM of internal standards CAPS, CHAPS, and PIPES was added to each replicate. The mixture was shaken at 4 °C for 30 min and centrifuged at 14,800× *g* for 10 min at 4 °C. Then, 100 µL of supernatant was transferred to the vials for metabolomic analysis and 10 µL was combined to make a pooled quality control (QC) sample.

For the lipidomics analysis, 240 µL of supernatant was transferred to microfuge tubes and evaporated at 20 °C under a stream of nitrogen and stored at −80 °C. On the day of analysis, the samples were dissolved in 80 µL of BuOH/MeOH/H_2_O (4.5:4.5:1, *v*/*v*), shaken for 30 min at room temperature, and sonicated for 1 h, keeping the temperature below 25 °C. The samples were centrifuged at 14,800× *g* for 10 min at 20 °C and then 70 µL of the sample was transferred to LC-MS vials and the leftover was combined to make a pooled QC sample.

### 4.6. Metabolomics LC-MS Data Acquisition

LC-MS data were acquired on a Q-Exactive Orbitrap mass spectrometer (Thermo Fischer Scientific, Waltham, MA, USA) coupled with high-performance liquid chromatography (HPLC) system Dionex Ultimate^®^ 3000 RS (Thermo Scientific, Waltham, MA, USA) [135] as outlined in the Figure 1. The samples were analysed as a single batch to avoid batch-to-batch variation and randomised to account for the LC-MS system drift over time. Chromatographic separation was performed on ZIC-pHILIC column (5 µm, 4.6 × 150 mm, SeQuant^®^, Merck) (Merck Millipore, Victoria, Australia) equipped with a guard (ZIC-pHILIC). The mobile phase (A) was 20 mM ammonium carbonate (Sigma Aldrich, New South Wales, Australia), and (B) acetonitrile (Thermo Fischer Scientific, Melbourne, Australia). The needle wash solution was 50% isopropanol. The gradient program started at 80% B and was decreased to 50% B over 15 min, then to 5% B until 18 min, kept at 5% B until 21 min, returned to 80% B by 24 min, and equilibrated at 80% B to 32 min. The flow rate was 0.3 mL.min^−1^ and column compartment temperature was 25 °C. The total run time was 32 min, with an injection volume of 10 µL. The mass spectrometer was operated in full scan mode with positive and negative polarity switching at 35 k resolution at 200 *m*/*z* with a detection range of 85 to 1275 *m*/*z*, AGC target 1 × 10^6^ ions, maximum injection time 50 ms. Heated electro-spray ionisation source (HESI) was set to 4.0 kV voltage for positive and negative mode, and sheath gas was set to 50, aux gas to 20, and sweep gas to 2 arbitrary units (AU), capillary temperature was 300 °C, and probe heater temperature was 120 °C.

### 4.7. Lipidomics LC-MS Data Acquisition

Chromatographic separation was performed on an Agilent Zorbax C8 (1.8 µm, 2.1 × 100 mm, Agilent Technologies, Victoria, Australia) equipped with a guard column (C8, 2 × 2 mm, Phenomenex, New South Wales, Australia) [136]. The mobile phase (A) was 40% isopropanol, 8 mM ammonium formate, and 2 mM formic acid, and (B) 98% isopropanol, 8 mM ammonium formate and 2 mM formic acid, and needle wash solution was 50% isopropanol. The gradient program started at 0% B and was increased stepwise to 20% B over 1.5 min, to 28% B over 5.5 min, to 35% B over 1 min, to 65% B over 16 min, and 100% B over 1 min. Wash at 100% B was continued for 2 min before decreasing to 0% B over the next 2 min, followed by equilibration at 0% B for 1 min. The flow rate was 0.2 mL.min^−1^ and column compartment temperature was 40 °C. The total run time was 30 min, with an injection volume of 10 µL. The mass spectrometer was operated in full scan mode with positive and negative polarity switching at 70 k resolution at 200 *m*/*z* with a detection range of 140 to 1300 *m*/*z*, AGC target 1 × 10^6^ ions, maximum injection time 50 ms. Heated electro-spray ionisation source (HESI) was set to 3.5 kV for both positive mode and negative modes, and sheath gas was set to 34 AU, auxiliary gas to 13 AU, and sweep gas to 1 AU. Capillary and probe heater temperatures were set to 250 and 190 °C, respectively.

### 4.8. Data Processing Using IDEOM

The acquired LC-MS data were processed in untargeted fashion using open source software IDEOM [137], which was initially used ProteoWizard to convert raw LC-MS files to mzXML format and XCMS to pick peaks. Mzmatch.R was used to convert to peakML files [138], and for sample alignment and the filtering of peaks using a minimum detectable intensity of 100,000, relative standard deviation (RSD) of <0.5 (reproducibility), and peak shape (codadw) of >0.8. Mzmatch was also used to retrieve missing peaks and annotate related peaks. Default IDEOM parameters were used to eliminate unwanted noise and artefact peaks. The loss or gain of a proton was corrected in negative and positive ESI mode, respectively, followed by putative identification of metabolites by accurate mass within 3 ppm mass error by searching against the Kyoto Encyclopedia of Genes and Genomes (KEGG), MetaCyc, and LIPIDMAPS databases. Additional manual curation was performed to remove putative lipids that did not elute at the expected retention time.

### 4.9. Data Analyses and Statistical Interpretation

We performed chemometric univariate and multivariate statistical analyses using the Metaboanalyst website (http://www.metaboanalyst.ca) [139]. Before chemometric univariate and multivariate statistical analyses, data integrity was checked and filtered to ensure all data had been included. The sample data (spectral data) were normalised, log transformed, and auto-scaled before analysis. For univariate analysis, volcano plot analysis was performed to identify differential metabolites using the t-test and fold-change (FC) methods, and plots log2 (fold-change > 2) on the *X*-axis against −log10 (*p*-value) from the t-test on the *Y*-axis. Benjamini–Hochberg correction or false discovery rate (FDR) was applied to compute the number of false positives out of significantly varied metabolic features.

In multivariate analyses, we performed principal component analysis (PCA) unsupervised method and hierarchical clustering analysis (HCA) with Euclidean measured distance, and the ward.D clustering algorithm was used to evaluate the difference in the concentration of each metabolite between sample groups.

Before the pathway analysis, IDs for the metabolites were obtained from the KEGG, LipidMAPS, PubChem Compound ID (PubChem CID), Human Metabolome Database (HMDB), and the Chemical Translation service (CTS; https://cts.fiehnlab.ucdavis.edu). Subsequently, we performed pathway enrichment analysis using the Metaboanalyst website (http://www.metaboanalyst.ca) [139].

### 4.10. Literature Review on Pharmacological Properties of Identified Metabolites

We conducted a comprehensive literature search for the pharmacological properties of metabolites identified in this study using various search engines, including PubMed, Medline, Google Scholar, and SciFinder Scholar. Keywords such as “anti-inflammatory,” “bioactivity,” “pharmacological activity,” and “anti-oxidant activity” were used to identify reported pharmacological activities of metabolites.

## 5. Conclusions

In summary, we show that the infective stages of two different STHs produce characteristic metabolites. The current study identified many unique metabolites (both polar as well as non-polar metabolites) present in the infective stages of *N. brasiliensis* (seven unique polar metabolites and 28 lipids in the somatic extract) and *T. muris* eggs (four unique lipids). Future studies should further characterise their identity and bioactivity in more detail. The vast array of metabolites identified from these two helminths’ infective stages could potentially serve as a database for the in-depth understanding of helminth biochemistry, which is currently lacking. Moreover, the suite of metabolites identified in this study presents a potential avenue for future research, particularly for the development of metabolite-based diagnosis tools and the identification of novel targets for anthelmintic drugs.

## Figures and Tables

**Table 1:** Polar metabolites (MSI-I identified) of infective stages of *N. brasiliensis* (L3) and *T. muris* (embryonated eggs).

Polar Metabolites	Formula ^a^	Mass (*m*/*z*)	Rt (min) ^c^	KEGG ID ^d^	Log_2_(FC) ^e^	Chemical Taxonomy ^b^	Reported Pharmacological Activities	Average Peak Area (mz/rt)		
								NB_SE	NB_ESP	TM_SE
Adenine	C_5_H_5_N_5_	135.054	8.32	C00147	5.23	6-aminopurines	Anti-inflammatory [32]	103,160,746	309,024	101,274
Adenosine	C_10_H_13_N_5_O_4_	267.096	8.23	C00212	5.21	Purine nucleosides	Anti-inflammatory [33]	327,057,005	0	347,270
Inosine	C_10_H_12_N_4_O_5_	268.080	10.32	C00294	4.77	Purine nucleosides	Anti-inflammatory [34,35]	14,631,676	0	18,472
l-Carnitine	C_7_H_15_NO_3_	161.105	12.32	C00487	4.56	Carnitines	Anti-inflammatory [36] and anti-oxidant [37]	81,471,762	1,911,162	125,132
Choline	C_5_H_13_NO	103.099	20.72	C00114	4.53	Cholines	Anti-inflammatory [38]	82,418,790	0	129,808
N(pi)-Methyl-l-histidine	C_7_H_11_N_3_O_2_	169.085	11.58	C01152	3.86	Histidine and derivatives	N/A	5,268,444	2,170,489	13,260
Xanthine	C_5_H_4_N_4_O_2_	152.033	11.08	C00385	3.62	Xanthines	Proinflammatory [39]	37,256,722	3,609,773	103,264
l-Aspartate	C_4_H_7_NO_4_	133.037	14.57	C00402	3.52	Aspartic acid and derivatives	Anti-inflammatory and neuroprotective [40,41]	3,886,437	402,353	9321
Succinate	C4H6O4	118.026	15.15	C00042	3.14	Dicarboxylic acids and derivatives	Activate inflammatory pathways [42,43]	29,148,318	5,130,431	99,396
5-Aminolevulinate	C_5_H_9_NO_3_	131.058	13.74	C00430	3.11	Delta amino acids and derivatives	Anti-inflammatory [44]	8,231,888	0	33,627
Adenosine 5′-monophosphate	C_10_H_14_N_5_O_7_P	347.063	13.03	C00020	3.05	Purine ribonucleoside monophosphates	Anti-inflammatory [45,46]	9,485,566	0	43,938
Hypoxanthine	C_5_H_4_N_4_O	136.038	9.56	C00262	2.84	Hypoxanthines	Anti-inflammatory and wound healing [47]	48,567,229	425,871	230,726
Lactose	C_12_H_22_O_11_	342.116	15.25	C00243	2.61	O-glycosyl compounds	N/A	94,498,840	420,550	552,215
l-Glutamate	C_5_H_9_NO_4_	147.053	14.24	C00025	2.30	Glutamic acid and derivatives	Antioxidant [48]	16,940,920	4,993,538	104,717
l-Methionine	C_5_H_11_NO_2_S	149.051	10.81	C00073	2.06	Methionine and derivatives	Anti-inflammatory [49] and antioxidant [50]	2,078,174	890,947	15,017
l-Histidine	C_6_H_9_N_3_O_2_	155.069	14.25	C00135	1.82	Histidine and derivatives	Anti-inflammatory [51]	14,964,799	1,089,738	135,916
4-Hydroxybenzoate	C_7_H_6_O_3_	138.031	10.37	C00156	1.73	Hydroxybenzoic acid derivatives	Neuroprotective [52]	1,566,927	1,019,203	13,620
l-Tyrosine	C_9_H_11_NO_3_	181.074	12.44	C00082	1.09	Tyrosine and derivatives	N/A	3,708,634	6,686,668	58,822
Deoxyadenosine	C_10_H_13_N_5_O_3_	251.101	7.48	C00559	−1.03	Purine 2′-deoxyribonucleosides	Cell growth inhibitor and cytotoxic [53]	628,485	0	39,096
(S)-Malate	C_4_H_6_O_5_	134.021	16.18	C00149	−1.28	Beta hydroxy acids and derivatives	N/A	51,344,999	43,880,026	0
D-Glucose 6-phosphate	C_6_H_13_O_9_P	260.029	15.83	C00092	−1.66	Hexose phosphates	N/A	402,720	0	42,982
2-Oxoglutarate	C_5_H_6_O_5_	146.021	15.77	C00026	−1.80	Gamma-keto acids and derivatives	Anti-inflammatory [54] and antioxidant [55]	249,067	2,843,652	0
l-Pipecolate	C_6_H_11_NO_2_	129.079	11.42	C00408	−1.94	l-alpha-amino acids	N/A	139,920	0	16,319
Mannitol	C_6_H_14_O_6_	182.079	13.30	C00392	−2.08	Sugar alcohol	Anti-edema [56]	299,808	0	37,781
l-Alanine	C_3_H_7_NO_2_	89.047	14.12	C00041	−2.27	l-alpha-amino acids	Anti-inflammatory [57,58,59]	3,102,359	3,529,005	0
Betaine	C_5_H_11_NO_2_	117.079	10.39	C00719	−2.39	Alpha amino acids	Neuroprotective [60]; improves intestinal barrier function [61]; hepatoprotective [62]; anti-inflammatory [38]	261,777,360	56,072,188	0
l-Lysine	C_6_H_14_N_2_O_2_	146.105	22.50	C00047	−2.45	l-alpha-amino acids	Anti-inflammatory [63,64]	2,260,735	1,941,102	0
l-Arginine	C_6_H_14_N_4_O_2_	174.111	24.05	C00062	−2.47	l-alpha-amino acids	Anti-inflammatory [65,66,67]	30,934,546	3,589,173	0
l-Glutamine	C_5_H_10_N_2_O_3_	146.069	14.44	C00064	−2.48	l-alpha-amino acids	Anti-inflammatory [57,58,59]	45,487,251	39,780,748	0
l-2-Aminoadipate	C_6_H_11_NO_4_	161.068	14.47	C00956	−2.49	l-alpha-amino acids	N/A	137,122	0	22,389
l-Phenylalanine	C_9_H_11_NO_2_	165.079	9.35	C00079	−2.50	l-alpha-amino acids	Anti-diabetic [68]	1,534,294	18,355,000	0
3’,5’-Cyclic AMP	C_10_H_12_N_5_O_6_P	329.052	8.72	C00575	−2.54	3′,5′-cyclic purine nucleotides	Anti-inflammatory [69]	281,873	0	57,179
N-Acetylputrescine	C_6_H_14_N_2_O	130.110	21.02	C02714	−2.58	Carboximidic acids	Lung cancer biomarker [70]	125,036	0	22,355
Thymine	C_5_H_6_N_2_O_2_	126.043	6.96	C00178	−3.62	Hydroxypyrimidines	N/A	169,388	0	64,297
l-Leucine	C_6_H_13_NO_2_	131.094	9.97	C00123	−4.14	Leucine and derivatives	Analgesic and anti-inflammatory [71,72]	1,593,314	10,121,672	735,972
Urocanate	C_6_H_6_N_2_O_2_	138.043	10.49	C00785	−4.16	Imidazolyl carboxylic acids and derivatives	Chemoattractant [73]	452,897	169,049	232,883
Azelaic acid	C_9_H_16_O_4_	188.105	10.69	C08261	−4.89	Medium-chain fatty acids	Anti-inflammatory [74]	131,028	874,645	116,635
Maleic acid	C_4_H_4_O_4_	116.011	12.18	C01384	−5.67	Dicarboxylic acids and derivatives	Inflammatory/Cytotoxic [75]	98,623	0	154,549
D-Glycerate	C_3_H_6_O_4_	106.026	11.85	C00258	ns	Sugar acids and derivatives	N/A	343,824	0	8833
Homogentisate	C_8_H_8_O_4_	168.042	9.42	C00544	ns	2(hydroxyphenyl)acetic acids	Pro-inflammatory [76,77]	0	640,416	0
Isocitrate	C_6_H_8_O_7_	192.027	19.04	C00311	ns	Tricarboxylic acids and derivatives	N/A	0	37,798,044	0
l-Citrulline	C_6_H_13_N_3_O_3_	175.095	14.84	C00327	ns	l-alpha-amino acids	Anti-inflammatory and antioxidant [78,79,80]	672,469	0	21,477
l-Proline	C_5_H_9_NO_2_	115.063	11.96	C00148	ns	Proline and derivatives	Anti-inflammatory [81]	9,760,345	1,106,588	269,125
l-Serine	C_3_H_7_NO_3_	105.042	15.56	C00065	ns	Serine and derivatives	Modulates adaptive immunity by controlling T cell proliferative capacity [82]; colon protection and mucosal healing [81]	337,344	45,878	6178
l-Threonine	C_4_H_9_NO_3_	119.058	14.14	C00188	ns	l-alpha-amino acids	Anti-inflammatory [83,84]	425,688	0	14,375
l-Tryptophan	C_11_H_12_N_2_O_2_	204.090	11.03	C00078	ns	Indolyl carboxylic acids and derivatives	Anti-inflammatory [85,86,87]	2,002,647	1,435,161	127,849
l-Valine	C_5_H_11_NO_2_	117.079	11.76	C00183	ns	Valine and derivatives	Anti-inflammatory [85]	474,387	9,518,904	25,791
Ll-2,6-Diaminoheptanedioate	C_7_H_14_N_2_O_4_	190.095	17.63	C00666	ns	Amino acid	N/A	0	115,608	0
N6,N6,N6-Trimethyl-l-lysine	C_9_H_20_N_2_O_2_	188.152	21.12	C03793	ns	l-alpha-amino acids	Cardiovascular disease biomarker [88]	462,527	208,659	12,965
Orotate	C_5_H_4_N_2_O_4_	156.017	10.27	C00295	ns	Pyrimidinecarboxylic acids	N/A	0	437,266	0
Pterin	C_6_H_5_N_5_O	163.049	10.30	C00715	ns	Pterins and derivatives	Biomarker of exercise-induced stress [89]	0	460,935	0
Pyridoxal	C_8_H_9_NO_3_	167.058	7.46	C00250	ns	Pyridoxals and derivatives	N/A	323,537	0	10,226
5-Oxoproline	C_5_H_7_NO_3_	129.042	9.82	C01879	ns	Alpha amino acids and derivatives	Promotes oxidative stress in neuropathology [90]	0	32,495,730	0
2,5-Dihydroxybenzoate	C_7_H_6_O_4_	154.026	8.30	C00628	ns	Hydroxybenzoic acid derivatives	Anti-cancer activity [91]	0	1,377,617	0
4-Trimethylammoniobutanoate	C_7_H_15_NO_2_	145.110	12.25	C01181	ns	Straight chain fatty acids	N/A	217,954	0	2264

^a^ Formula; ^b^ Chemical taxonomy = Formula and chemical taxonomy for compounds were taken from human metabolome database (HMDB, http://www.hmdb.ca); ^c^ Rt = retention time in minutes; ^d^ KEGG ID (http://www.genome.jp/kegg/) contains information on biosynthetic and metabolic pathways of identified compounds; ^e^ log 2(FC) is a fold change between NB_SE and TM_SE; Abbreviations: ns = not significant; ID = identity; NB_SE = the somatic extract of infective third-stage larvae (L3) of *N. brasiliensis*; NB_ESP = the excretory/secretory products (ESP) of *N. brasiliensis* L3; TM_SE = the somatic extract of *T. muris* embryonated eggs. Note: peak areas values of media were subtracted from the samples.

**Table 2:** Putative lipids unique to infective stages of *N. brasiliensis* (L3) and *T. muris* (embryonated eggs).

Putative Lipids	Formula ^a^	Mass (*m*/*z*)	Rt (min) ^c^	Chemical Taxonomy ^b^	LipidMAPS ID ^d^	Peak Areas (mz/rt)		
						NB_SE	NB_ESP	TM_SE
DG(41:7)	C_44_H_72_O_5_	680.536	16.05	Glycerolipids	LMGL02010545	124,404	0	0
FA hydroxy(12:0) dodecanoic acid	C_12_H_24_O_3_	238.155	2.19	Fatty Acyls	NA	0	0	51,014
LacCer(d38:0)	C_50_H_97_NO_13_	919.696	18.98	Sphingolipids	LMSP05010122	139,285	0	0
LacCer(d40:0)	C_52_H_101_NO_13_	947.727	20.22	Sphingolipids	LMSP05010124	182,175	0	0
LysoPE(22:2)	C_27_H_52_NO_7_P	533.350	4.84	Glycerophospholipids	LMGP02050024	136,295	0	0
PE-Cer(d40:1)	C_42_H_85_N_2_O_7_P	760.610	13.49	Sphingolipids	LMSP03020086	117,114	0	0
PE-Cer(d38:1)	C_40_H_81_N_2_O_7_P	732.579	12.26	Sphingolipids	LMSP03020064	112,168	0	0
PA(25:0)	C_28_H_55_O_8_P	550.364	12.05	Glycerophospholipids	LMGP10010001	0	0	374,621
PA(26:0)	C_29_H_57_O_8_P	564.379	12.58	Glycerophospholipids	LMGP10010980	0	0	1,921,006
PC(36:7)	C_44_H_74_NO_8_P	775.514	10.73	Glycerophospholipids	LMGP01012100	105,372	0	0
PC(P-32:2)	C_40_H_76_NO_7_P	713.536	13.07	Glycerophospholipids	NA	657,868	0	0
PC(P-36:2)	C_44_H_84_NO_7_P	769.599	14.95	Glycerophospholipids	LMGP01030137	138,297	0	0
PE(28:2)	C_33_H_62_NO_8_P	631.422	7.68	Glycerophospholipids	LMGP02011238	127,324	0	0
PE(48:2)	C_53_H_102_NO_8_P	911.734	20.75	Glycerophospholipids	LMGP02010893	466,471	0	0
PE(40:5)	C_45_H_80_NO_8_P	815.542	12.76	Glycerophospholipids	LMGP02010893	483,243	0	0
PE(48:1)	C_53_H_104_NO_8_P	913.751	21.37	Glycerophospholipids	NA	1,154,195	0	0
PE(O-20:0)	C_25_H_54_NO_6_P	495.370	7.71	Glycerophospholipids	LMGP02060005	108,008	0	0
PE(P-20:0)	C_25_H_52_NO_6_P	493.354	7.86	Glycerophospholipids	LMGP02070004	375,557	0	0
PE(P-36:4)	C_41_H_74_NO_7_P	723.521	12.51	Glycerophospholipids	LMGP02030093	195,178	0	0
PE(P-36:5)	C_41_H_72_NO_7_P	721.504	11.93	Glycerophospholipids	LMGP02030028	87,036	0	0
PE(P-38:6)	C_43_H_74_NO_7_P	747.519	12.36	Glycerophospholipids	LMGP02030001	308,137	0	0
PG(36:1)	C_42_H_81_O_10_P	776.557	11.98	Glycerophospholipids	LMGP04010037	699,995	0	0
PI(37:6)	C_46_H_77_O_13_P	868.512	9.51	Glycerophospholipids	LMGP06010790	119,218	0	0
PI(38:7)	C_47_H_77_O_13_P	880.512	9.44	Glycerophospholipids	LMGP06010792	168,877	0	0
PI(P-37:2)	C_46_H_85_O_12_P	860.576	11.78	Glycerophospholipids	LMGP06030067	94,153	0	0
PS(28:2)	C_34_H_62_NO_10_P	675.412	6.97	Glycerophospholipids	LMGP03010919	253,811	0	0
PS(36:4)	C_42_H_74_NO_10_P	783.506	10.48	Glycerophospholipids	LMGP03010038	141,937	0	0
PS(36:5)	C_42_H_72_NO_10_P	781.491	10.06	Glycerophospholipids	LMGP03010654	115,385	0	0
PS(O-38:0)	C_44_H_88_NO_9_P	805.621	15.40	Glycerophospholipids	LMGP03020051	86,582	0	0
PS(O-34:0)	C_40_H_80_NO_9_P	749.558	12.46	Glycerophospholipids	LMGP03020043	0	0	1883
SM(d41:2)	C_46_H_91_N_2_O_6_P	844.667	14.55	Sphingolipids	LMSP03010074	29,023	0	0
SM(d42:2)	C_47_H_93_N_2_O_6_P	812.676	15.24	Sphingolipids	LMSP03010007	78,211	0	0

^a^ Formula; ^b^ Chemical taxonomy = Formula and chemical taxonomy for compounds were taken from LipidMAPS database (https://www.LipidMAPS.org); ^c^ Rt = retention time in minutes; ^d^ LIPIDMAPS ID (https://www.LipidMAPS.org) contains information on biosynthetic and metabolic pathways of identified lipids; Abbreviations: ID = identity; NB_SE = the somatic extract of infective third-stage larvae (L3) of *N. brasiliensis*; NB_ESP = the excretory/secretory products (ESP) of *N. brasiliensis* L3; TM_SE = the somatic extract of *T. muris* embryonated eggs.

## Supplementary Materials

The following are available online at https://www.mdpi.com/2218-1989/10/11/446/s1, Table S1: Total putative polar metabolites identified in the infective stages of *Nippostrongylus brasiliensis* and *Trichuris muris*, Table S2: Total putative lipids identified in the infective stages of *Nippostrongylus brasiliensis* and *Trichuris muris*, Table S3: Fold change analysis of putative lipids between the infective stage of *N. brasiliensis* somatic extract (NB_SE) and *T. muris* embryonated egg extract (TM_SE).
